# Radio-Frequency/Microwave Gas Sensors Using Conducting Polymer

**DOI:** 10.3390/ma13122859

**Published:** 2020-06-25

**Authors:** Chorom Jang, Jin-Kwan Park, Gi-Ho Yun, Hyang Hee Choi, Hee-Jo Lee, Jong-Gwan Yook

**Affiliations:** 1 Department of Electrical and Electronic Engineering, Yonsei University, Seoul 03722, Korea; chorom@yonsei.ac.kr (C.J.); paladin91@yonsei.ac.kr (J.-K.P.); 2 Department of Information and Communications Engineering, Sungkyul University, Gyeonggi-Do 14097, Korea; ghyun@sungkyul.ac.kr; 3 Institute of Engineering Research, Yonsei University, Seoul 03722, Korea; netchoi@yonsei.ac.kr; 4 Department of Physics Education, College of Education, Daegu University, Gyeongsan 38453, Korea; hjlee@daegu.ac.kr

**Keywords:** radio-frequency, microwave, conducting polymer, organic material, gas sensor, polyaniline, polypyrrole, poly(3,4-ethylenedioxythiophene)

## Abstract

In this review, the advances in radio-frequency (RF) /microwave chemical gas sensors using conducting polymers are discussed. First, the introduction of various conducting polymers is described. Only polyaniline (PANi), polypyrrole (PPy) and poly(3,4-ethylenedioxythiophene) (PEDOT), which are mainly used for gas sensors in RF/microwave region, are focused in this review. Sensing mechanism of the three conducting polymers are presented. And the RF/microwave characteristics and RF/microwave applications of the three conducting polymers are discussed. Moreover, the gas sensors using conducting polymers in RF/microwave frequencies are described. Finally, the the challenges and the prospects of the next generation of the RF/microwave based chemical sensors for wireless applications are proposed.

## 1. Introduction

**Figure 1:**
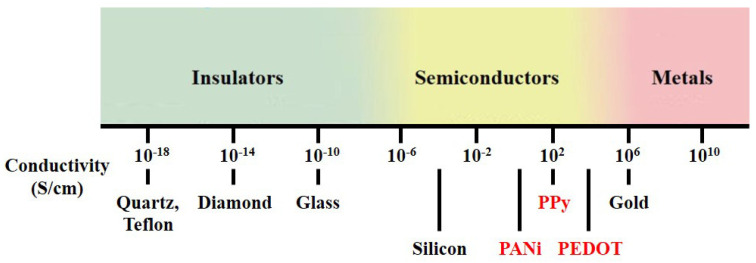
Conductivity range of conducting polymer.

Polymers possess many advantages over other materials, as various chemical structures and adjustable surface functionalities are exploitable. Among synthetic polymers, conducting polymers (CPs) have received considerable attention since the initial discovery of polyacetylene in the late 1970s [1]. Unlike conventional polymers, which contain only single bonds (σ) and are not conductive, CPs form a conjugated structure consisting of a series of alternating single and double bonds (π) along the polymer chain, which provides semiconducting properties. CPs constitute a class of organic materials with conductive properties similar to those of semiconductors, and the conductivity range of CPs is located between those of insulators and metals, as shown in Figure 1. The unique chain structure of CPs allows precise control of conductivity according to specific applications. The molecular chain structure of CPs can be conveniently modified by copolymerization or structural derivations. Moreover, CPs also exhibit various extraordinary properties, such as low cost, chemical reactivity at room temperature, low redox potential and good processability [2]. As a result, CPs are attractive as sensing materials for various gas sensor applications.

There are various conducting polymers such as polyaniline (PANi), polypyrrole (PPy), poly(3,4-ethylenedioxythiophene) (PEDOT), polythiophene (PT), polyacetylene (PA), poly(p-phenylene vinylene) (PPv), poly(p-phenylene) (PPP), Poly(p-phenylene sulfide) (PPS) and Polyisothianaphthene (PITN) as shown in Table 1. However, only PANi, PPy and PEDOT, which are mainly used for gas sensors in the RF/microwave regime, are discussed in this review. Three CPs can be distinguished by chemical structure. All CPs have aromatic rings and π electron conjugation. The alternating single and double bonds of these CPs create broad π electron conjugation, which decreases the energy and stabilizes the molecule. However, PPy has a nitrogen atom in the aromatic ring, and PANi has a nitrogen atom outside the aromatic ring. PEDOT has a sulfur atom in the aromatic ring.

The electrons localized in the σ bonds in the CP chain are very capable of stabilizing and electrically insulating the polymer. However, the electrons in the π bonds are delocalized along the chain and easily removed from the bond, conferring electrical properties. Therefore, CP requires doping at conjugated double bonds to achieve high electrical conductivity, which is analogous to the semiconductor doping process. PPy and PEDOT can be doped by a redox reaction, and PANi can be doped by protonation.

**Figure 2:**
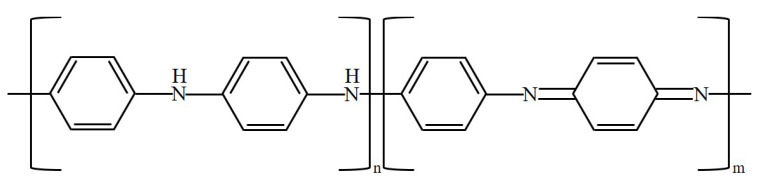
Oxidation of PANi.

The oxidation process of PANi is illustrated in Figure 2 [3]. The conductivity of PANi can be modulated through the oxidation state. PANi has three main states: leucoemeraldine, emeraldine, and pernigraniline. As oxidation progresses, PANi progresses from the leucoemeraldin state to the emeraldine state and then to the pernigranilin state. In the leucoemeraldine state, n = 1 and m = 0, as shown in Figure 2, and the conductivity is low. In the emeraldine state, n = 0.5 and m = 0.5, and the conductivity is the highest. In the pernigranilin state, n = 0 and m = 1, and the conductivity is low.

**Figure 3:**
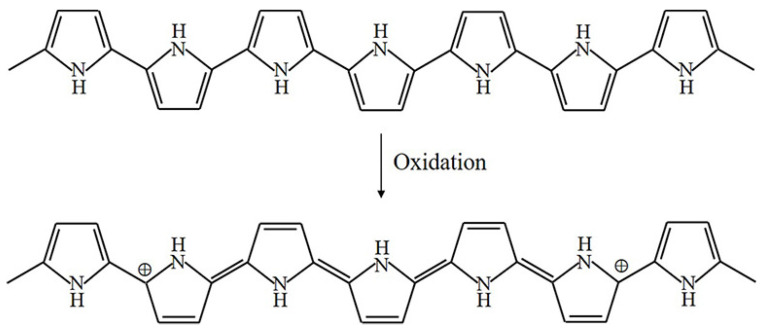
Oxidation of PPy.

**Figure 4:**
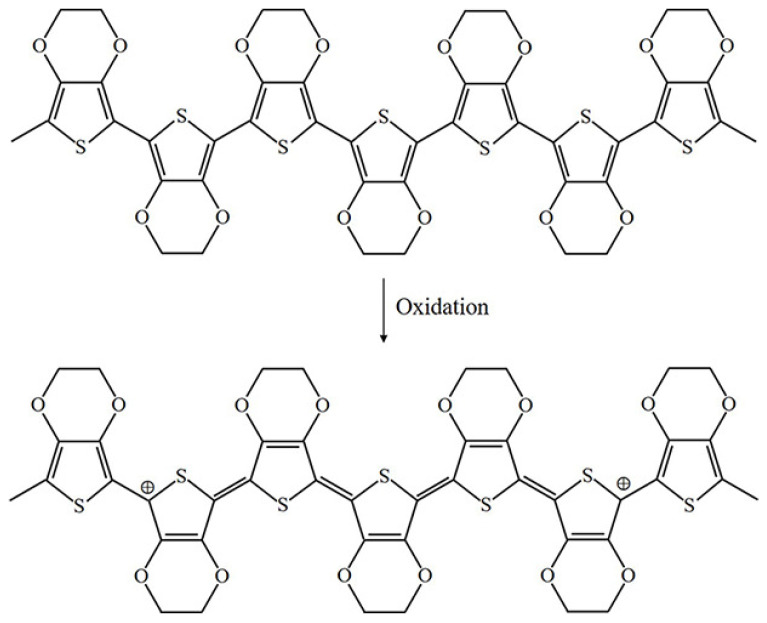
Oxidation of PEDOT.

The oxidation process of PPy is illustrated in Figure 3 [4]. Upon oxidation, π electrons in the conjugated bond are removed, yielding local relaxation of the benzoid structure towards the quinoid structure, which creates a pair of radicals and a positive charge. Further oxidation results in the formation of bipolarons; single and double bonds replace each other, and two cations remain in the PPy structure. Thus, the cation formed in PPy can transfer through the π electron cloud to yield electronic conductivity. Moreover, the oxidation process of PEDOT is similar to that of PPy, as illustrated in Figure 4.

## 2. Conducting Polymers for Chemical Gas Detection

### 2.1. Sensing Mechanism of the PPy

**Figure 5:**
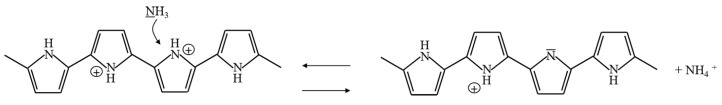
Sensing mechanism of PPy exposed to carbon ammonia.

PPy can be utilized as a sensing material for the detection of NH${}_{3}$, H${}_{2}$S, NO${}_{2}$ and I${}_{2}$ by redox reactions. PPy undergoes electron transfer, and changes in resistance, conductance and work function occur. The process of electron transfer is different for different gases. When PPy reacts with electron acceptors such as NO${}_{2}$, H${}_{2}$S and I${}_{2}$, electrons in the aromatic rings of PPy are removed, and the electric conductance of PPy is enhanced [5,6,7,8]. On the other hand, when PPy reacts with NH${}_{3}$, which is an electron donor, PPy obtains electrons from NH${}_{3}$, and the electric resistance decreases rapidly [9,10,11]. In particular, the resistance of PPy is recovered after exposure to N${}_{2}$ or air. The reaction processes of adsorption and desorption can be expressed as shown in Equation (1) and Figure 5 [12,13,14]:

(1) $$PPy^{+}+\ddot{N}H_{3}⇄PPy^{+}{\left(-H\right)}^{0}+\ddot{N}H_{4}^{+}$$

### 2.2. Sensing Mechanism of the PANi

**Figure 6:**
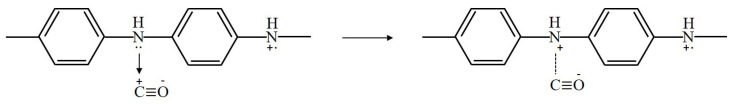
Sensing mechanism of PANi exposed to carbon monoxide.

PANi also reacts with gases by a redox reaction, but PANi has characteristics of partial charge transfer. The direction of partial charge transfer is determined by the work function of the conducting polymer and the electronegativity of the gas vapor [15]. The partial charge transfer consists of electron transfer and proton transfer. When PANi is exposed to carbon monoxide (CO), the electrons in the PANi film are removed, and the resistance of PANi is decreased [16,17]. The mechanism by which PANi senses CO can be expressed as shown in Figure 6.

A lone pair of electrons on the amine nitrogen (-NH-) is removed by the stable structure of C${}^{+}$≡ O${}^{-}$ with a positive charge on the carbon atom. This positive charge on the carbon atom is transferred to amine nitrogen. Then, the positive charge carriers are increased on the PANi backbone, and the conductivity is increased.

**Figure 7:**
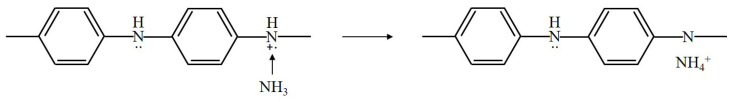
Sensing mechanism of PANi exposed to ammonia.

PANi has specificity in that the doped state is controlled by an acid/base reaction. Thus, PANi is widely utilized for the detection of acidic and basic gases. The sensing mechanism of PANi exposed to NH${}_{3}$ is shown in Figure 7.

The proton on the amine nitrogen is transferred to the NH${}_{3}$ molecule to form ions, and PANi is converted into its base form. When ammonia is removed, the ammonium ion is decomposed to NH${}_{3}$ and a proton. Therefore, the reaction process is reversible [18,19]. Additionally, PANi undergoes proton transfer when exposed to acidic gases such as H${}_{2}$S and HCl [20,21]. When H${}_{2}$ is adsorbed onto the positively charged nitrogen atom of PANi, the resistance of PANi is decreased due to the formation fo a new N-H bond between the hydrogen and nitrogen atoms [22].

### 2.3. Sensing Mechanism of the PEDOT

PEDOT exhibits high stability and high conductivity in its oxidized state. However, to improve the characteristics of PEDOT, PEDOT is usually coupled with poly(styrene sulfonate) (PSS). The combination of two cations of PEDOT with negatively charged PSS anions formed PEDOT:PSS. PEDOT:PSS is soluble in water and has improved conductivity and stabilization.

PEDOT:PSS can be utilized as a sensing material for the detection of ethanol gas, alkane gas, and water vapor by redox reactions. PEDOT:PSS undergoes electron transfer and changes in resistance depending on the specific gas. When PEDOT:PSS reacts with electron donors, such as ethanol gas [23] and alkane gas [24], the electric conductance of PEDOT:PSS is enhanced. On the other hand, when PEDOT:PSS reacts with water vapor [25,26], which is an electron acceptor, the electric conductance of PEDOT:PSS is enhanced. The resistance of PEDOT:PSS is removed after exposure to N${}_{2}$ or air. The reaction process of ethanol gas and alkane gas can be expressed as follows:

(2) $$\begin{array}{cc} \; & O_{2}\left(gas\right)\to O_{2}\left(phys\right)\to O_{2}^{-}\left(chem\right)\\ \; & C_{2}H_{5}OH\to CH_{3}CHO+H_{2}\\ \; & 2CH_{3}CHO\left(ad\right)+5O_{2}^{-}\to 4CO_{2}+4H_{2}O+5e^{-} \end{array}$$

(3) $$C_{n}H_{2n+2}+2O^{-}\to C_{n}H_{2n}O+H_{2}O+e^{-}$$

The reaction process of the water vapor can be expressed as follows:

(4) $$\begin{array}{c} H_{2}O+PSS\left(HSO_{3}\right)\to H_{3}O^{+}+PSS\left(SO_{3}^{-}\right) \end{array}$$

## 3. Concept of Radio-Frequency/Microwave-Based Chemical Gas Sensor

### 3.1. Radio-Frequency/Microwave Sensing Parameters

In the RF/microwave regime, many sensors are based on various devices from passive components to active components. These microwave sensors are characterized by the operating frequency and/or the S-parameter, which is defined as the ratio of the voltage of the output port to the voltage of the input port when all other ports are terminated in matched loads, as follows:

(5) $$S_{\mathrm{oi}}=\frac{V_{o}^{-}}{V_{i}^{+}}{|}_{V_{k}^{+}=0,k\neq i}.$$

When *i* is equal to *o*, $S_{ii}$ is the reflection coefficient from port *i*. Otherwise, when *i* is not equal to *o*, $S_{oi}$ represents the transmission coefficient from port *i* to port *o*. The S-parameter can be converted to other parameters, such as impedance, admittance, and the ABCD parameter. The S-parameter is expressed in different forms, such as magnitude and phase, real and imaginary.

Owing to their cost effectiveness, reusability and low power consumption as well as the multiparameter measurement, microwave sensors have been used in various applications for decades. Microwave sensors detect an analyte by variations in their characteristics, such as operating frequency, reflection coefficient, transmission coefficient, and phase. Park et al. [27] detected both heart rate and breath rate by the variations in the impedance of the resonator in response to periodic movements of the human lungs and heart. Jang et al. [28,29] reported a fluidic glucose sensor that detects the concentration of 100 mg/dL intervals from 0 to 400 mg/dL with the variation in the transmission coefficient of the resonator. Lee et al. [30] detected 1-μℓ phosphate-buffered saline (PBS) droplets by changes in the resonance frequency, transmission coefficient and Q-factor. Lee et al. [31] extracted the vital signs of multiple targets by using the phase information of the frequency modulation continuous wave radar.

### 3.2. Radio-Frequency/Microwave Characteristics of Conducting Polymers

To utilize the advantages of conducting polymers for microwave devices, various studies have been reported to analyze the microwave characteristics of conducting polymers. Although there are numerous conducting polymers, this review addresses the RF/microwave characteristics of PANi, PPy and PEDOT.

#### 3.2.1. Radio-Frequency/Microwave Characteristics of the PANi

The conducting polymer PANi has the advantages of environmental stability and ease of synthesis. Thus, the microwave characteristics of PANi doped with various composites have been analyzed. Wang et al. [32] analyzed the electric dielectric loss $tan\delta_{e}$ and magnetic dielectric loss $tan\delta_{m}$ of (ES)/γ-Fe${}_{2}$O${}_{3}$, which is protonated PANi synthesized by different γ-Fe${}_{2}$O${}_{3}$ contents (10%, 20%, and 30%). They also found that (ES)/γ-Fe${}_{2}$O${}_{3}$ has the potential of a microwave absorption material through reflection loss. Abbas et al. [33] analyzed the electric dielectric and reflection loss of PANi synthesized with different ratios of BaTiO${}_{3}$, which is a ferroelectric material with high resistivity. BaTiO${}_{3}$ and PANi were prepared in three ratios (75:25, 50:50, and 25:75), and these samples were measured at microwave frequencies in the X-band (from 8.2 to 12.4 GHz). They found that dc/ac conductivity, dipole relaxation and reflection loss increased with increasing PANi content. They also found that BaTiO${}_{3}$-PANi could be used as an absorber and that the matching frequency decreased with increasing thickness of the BaTiO${}_{3}$-PANi. Gandhi et al. [34] measured the relative permittivity and relative permeability of PANi synthesized with cobalt ferrite (CoFe${}_{2}$O${}_{4}$) in the Ku-band (from 12.4 to 18 GHz). The ratio of PANi:CoFe${}_{2}$O${}_{4}$ was 2:1, 1:1, 1:2 and 1:3. They analyzed the EMI shielding effectiveness (SE), which is defined as the ratio of transmitted power to incident power. The SE of each sample was analyzed by dividing by SE due to absorption by the SE due to reflection. They found that the SE of PANi-CoFe${}_{2}$O${}_{4}$ is mainly due to absorption and that the SE of PANi-CoFe${}_{2}$O${}_{4}$ increases with increasing content of CoFe${}_{2}$O${}_{4}$.

#### 3.2.2. Radio-Frequency/Microwave Characteristics of the PPy

PPy is widely studied due to its ease of preparation, chemical and thermal stability, inherent electrical conductive properties, aqueous solubility, and variable surface properties [35,36]. Epron et al. [37] measured the dielectric constants of polypyrrole synthesized in aqueous medium with various stabilizers and oxidizing agents. The stabilizers used in this paper are methylcellulose, 88% hydrolysed poly(vinyl acetate) and 2-hydroxyethylcellulose. The oxidizing agents used are FeCl${}_{3}$·t6H${}_{2}$O, Fe(ClO${}_{4}$)${}_{3}$·xH${}_{2}$O, CuBr${}_{2}$ anh., and Fe(NO${}_{3}$)${}_{3}$·9H${}_{2}$O. The dielectric constants are measured by a cavity resonant frequency shift at 5.03 GHz. They found that the dielectric constant is increased with the concentration of PPy particles. Additionally, the best oxidizing agent to achieve the best conductivity is FeCl${}_{3}$·6H${}_{2}$O when the PPy is stabilized by methylcellulose.

The dielectric constants as a function of PPy dopant concentration from 0.02 M to 1 M are reported by Kaynak et al. [38]. They measured the dielectric constants by the changes in the cavity resonance frequency and Q factor of the cylindrical and rectangular cavity operating at 2.45 GHz and 10 GHz. They found that both the dielectric constant and the dielectric loss increased with increasing PPy dopant concentration. They also measured the complex dielectric constant of PPy films as a function of temperature from 90 K to 300 K with different dopant levels. They also studied the conductivity, transmission, reflection and absorption of PPy films [39]. The microwave transmission and reflection characteristics of the PPy films were measured using a vector network analyzer and rectangular to coaxial transducers at 2.45 GHz and 10 GHz. The conductivity and reflection coefficient of the PPy films increased with increasing PPy doping levels. The transmission decreased with increasing PPy doping levels. The microwave absorption increased with increasing doping levels, and the maximum value occurred at intermediate doping levels where there was a maximum rate of variation in reflection and transmission and then decreased with the doping levels thereafter. Additionally, the transmission decreased and the reflection increased with increasing thickness of the PPy films from 50 microns to 1000 microns.

#### 3.2.3. Radio-Frequency/Microwave Characteristics of the PEDOT

PEDOT has attracted attention due to its high conductivity and environmental stability [40,41]. Ni et al. [42] studied the microwave properties of PEDOT hollow microspheres synthesized by 4.5 × 10${}^{-2}$ M 3,4-ethylene dioxythiophene (EDOT). The complex permittivity and complex permeability were measured by a network analyzer from 2 GHz to 18 GHz. The return loss was calculated using measured data. As a result, the real and imaginary parts of the complex permittivity decreased with increasing frequency. However, the real part of the complex permeability remained almost 1. The PEDOT hollow microsphere had no magnetic loss from 2 GHz to 18 GHz. They also found that the thickness of the PEDOT layer affects the return loss. The layer thicknesses under the test were 1, 2, 3 and 4 mm. The frequency and magnitude of the return loss at peak decreased with increasing layer thickness. Tamburri et al. [43] characterized PEDOT synthesized with polystyrene sulfonate (PSS) using the relationship between the reflection coefficient and the admittance of the sample. They investigated the admittance of the PEDOT-PSS from 40 MHz to 40 GHz. Then, the conductivity of the sample was derived from the admittance. Kang et al. [44] analyzed a thin PEDOT:PSS film from 1 GHz to 10 GHz. The weight ratio of the PEDOT:PSS was 1:2.5. The thin PEDOT:PSS film was deposited over the microstrip line, and the reflection and transmission coefficients were measured using the vector network analyzer. Then, the transmission line parameters (resistance, inductance, capacitance, conductance) were extracted using a de-embedding technique of thru-reflect-line (TRL) calibration.

Lakshmi et al. [45] compared conducting polymers such as PANi, PPy and PEDOT in powder form. The dielectric constant, dielectric loss factor, absorption coefficient, heating coefficient, conductivity, tangent loss and penetration depth were analyzed from 2 GHz to 4 GHz (S band). The microwave properties of the conducting polymers in powder form were investigated by the cavity perturbation method. The cavity resonant frequency and Q factor of the empty cavity and the loaded cavity were measured. The microwave properties were calculated from measured data. As a result, the dielectric constant, dielectric loss factor, absorption coefficient, conductivity and tangent loss of the conducting polymers follow the order PEDOT > PANi > PPy. However, the heating coefficient and penetration depth of the conducting polymers follow the order PPy > PANi > PEDOT.

### 3.3. Radio-Frequency/Microwave Applications Using Conducting Polymers

Due to the good electrical conductivity, dielectric properties, simple and effective surface coating, coating thickness control, compatibility with other polymers, transparency and processibility of the conducting polymers, they are widely used as shielding and absorbing materials in the microwave region [46,47].

Electromagnetic interference (EMI) is a severe problem. EMI can cause malfunction of electric devices and affect various lives. Thus, shielding materials are essential for the reduction of EMI. EMI shielding is defined as the sum of reflection, absorption and multiple reflection loss at the interfaces [48,49]. It has been reported that nanostructured PANi [50], PANi microtubes (PS) [51] and PANi/polystyrene (PS) can be used as EMI shielding materials at 12.4–18 GHz, 1–18 GHz and 11.6–17.6 GHz, respectively. A reduced graphene oxide (RGO) and PMMA composite film can also be used as a shielding material [52]. The shielding effectiveness increases with increasing RGO content and film thickness. The reflection shielding effectiveness is increased by utilizing electrical conducting fillers such as multiwalled carbon nanotubes (MWCNTs), metals, and graphene because the metallic islands inside the conducting polymers are bridged by conducting filler particles [53,54,55,56,57,58].

There are various studies on conducting polymers for military applications. To block the detection of aircraft during war, the radar cross section (RCS) should be reduced, which can be performed by using radar absorbing materials (RAM). Currently, RAM is receiving attention due to its various applications, such as aerospace and aeronautics, electromagnetic protection from natural phenomena such as lightning, high-intensity radiated field protection, and human exposure mitigation [59]. The ideal RAM should have two properties. One is that the intrinsic impedance of the RAM is equal to the impedance of free space. The other is that the electromagnetic wave in the RAM is rapidly attenuated [60]. Due to the characteristics in the microwave region, conducting polymers can be used as the RAM. An ultra-thin radar absorber using a metal textile doped with PPy at 3.88–5.88 GHz [61], a PPy and polyurethane mixture at 8–12 GHz [62], paint/rubber containing PPy powder and PPy-coated structural phenolic foams at 12–18 GHz [63], and PPy grown on cellulose at 2–18 GHz [64] were verified as the RAM. PANi doped with two acids in the X-band [65], barium strontium titanate (BST) and expanded graphite (EG) encapsulated in the PANi matrix at 12.4–18 GHz [66], the electromagnetic bandgap (EBG) based on PEDOT [67], and PEDOT with polyurethane binder in the X-band [68,69] also show good absorbing performance. In particular, PEDOT can be used as a frequency selective surface (FSS) [70] and stealth technology [71] in the X-band.

## 4. Radio-Frequency/Microwave Chemical Gas Sensors Using Conducting Polymers

### 4.1. PANi-Based Radio-Frequency/Microwave Chemical Gas Sensors

**Figure 8:**
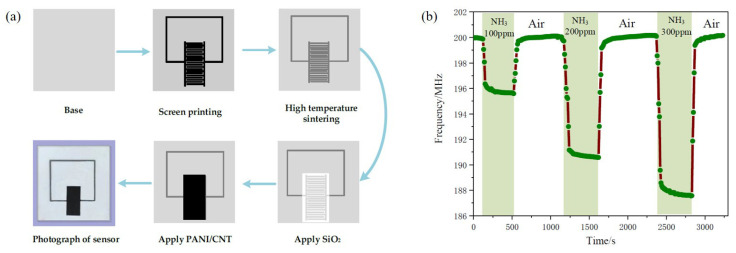
Characterization of the PANi/CNT composite-based ammonia gas sensor. (**a**) Fabrication process of the sensor. (**b**) Response and recovery time curve with different concentrations of ammonia gas [72].

PANi is widely utilized for various gas sensors at radio frequencies. Shen et al. proposed a wireless passive gas sensor based on LC mutual coupling [72]. The interdigitated electrode-based LC resonator is designed to experience resonance at 213.6 MHz. PANi was synthesized with CNTs, and the PANi/CNT composite was located on the interdigitated electrode. The PANi/CNT-based gas sensor was fabricated as shown in Figure 8a. When the sensor was exposed to ammonia gas, the capacitance of the interdigitated electrode was changed, and a resonance frequency shift occurred. When the sensor was exposed to 100, 200 and 300 ppm ammonia gas, the resonance frequency shifts were 4.352 MHz, 9.235 MHz and 12.070 MHz, respectively, as shown in Figure 8b. It was demonstrated that the sensitivity of the PANi/CNT-based sensor is approximately 0.04 MHz/ppm under a concentration of 300 ppm, and the variation in the resonant frequency is large at a low concentration of ammonia gas.

To detect gas using PANi, a surface acoustic wave (SAW) sensor is widely utilized. SAW sensors are suitable for gas sensing due to their high sensitivity, fast and reliable response, potential for wireless sensing, small size and low power consumption [73]. A shear horizontal surface acoustic wave (SH-SAW) sensor coated with PANi film was proposed to detect the presence of ammonia gas [74]. The proposed SH-SAW sensor was operated at 148 MHz. The sensor detected concentrations of ammonia gas from 20 ppm to 70 ppm by the frequency shift. They also presented a Rayleigh surface acoustic wave (RSAW) sensor with a copper-doped PANi-SnO${}_{2}$ nanocoposite thin film to detect the presence of nitric oxide (NO) at room temperature [75]. The RSAW was operated at 98.47 MHz, and the sensor detected NO from 100 ppb to 350 ppb by the frequency shift. Shi et al. also proposed the SAW-based gas sensor at 146 MHz [76]. Palladium-phthalocyanine (PdPc)-PANi was utilized as the sensing material for the detection of phosphorus-containing toxic gas. When the sensor was exposed to dimethyl methylphosphonate (DMMP), it exhibited a sensitivity of 550 kHz/ppm. Wang et al. proposed the SAW sensor with a graphene/PANi nanocomposite for NO detection by the frequency shift at 64.9 MHz [77]. It was demonstrated that the sensor exhibited an almost linear frequency shift for NO concentrations from 1 to 50 ppm. The sensitivity of the graphene/PANi composite was much higher than that of the PANi or graphene sensor. Sadek et al. proposed the PANi/tungsten trioxide (WO${}_{3}$) nanofiber composite-based SAW sensor for detection of hydrogen (H${}_{2}$) by the frequency shift at 107.2 MHz [78]. The range of H${}_{2}$ was from 0.06% to 1%. It was demonstrated that the sensor response was 7 kHz with 1% of H${}_{2}$ and that the sensor exhibits good stability. They also studied the PANi/In${}_{2}$O${}_{3}$-based SAW sensor for the detection of H${}_{2}$, carbon monoxide (CO), and nitrogen dioxide (NO${}_{2}$) by the frequency change [79]. The sensor response was 11 kHz, 2 kHz and 2.5 kHz toward 1% of H${}_{2}$, 500 ppm of CO and 2.12 ppm of NO${}_{2}$, respectively. It was verified that PANi/IN${}_{2}$O${}_{3}$ is more sensitive to H${}_{2}$ than PANi/WO${}_{3}$.

### 4.2. PPy-Based Radio-Frequency/Microwave Chemical Gas Sensors

**Figure 9:**
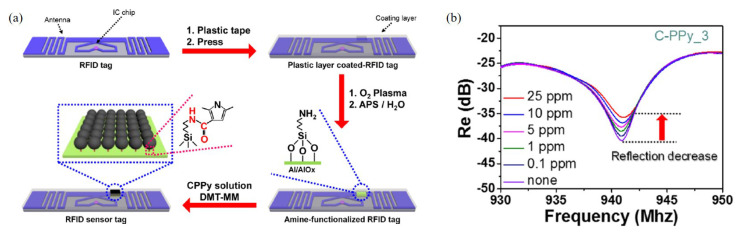
Characterization of the RFID-based ammonia gas sensor with C-PPy. (**a**) Schematic of the RFID sensor tag with C-PPy nanoparticles in the desired position. (**b**) Change in the reflection coefficient of the C-PPy-based RFID sensor tag. (Reprited with permission from [80]. Copyright 2016 American Chemical Society).

PPy is another gas sensing material synthesized with various materials. Jun et al. designed a radio frequency identification (RFID)-based wireless sensor using carboxyl group-functionalized PPy (C-PPy) nanoparticles at 940 MHz [80]. The C-PPy nanoparticles are utilized as the sensing material for the detection of ammonia gas and deposited on the RFID sensor tag, as shown in Figure 9a. When the sensor was exposed to ammonia gas ranging from 0.1 ppm to 25 ppm, the reflection coefficient was as shown in Figure 9b. The sensor exhibited ultrahigh sensitivity to ammonia, detecting concentrations as low as 0.1 ppm. Additionally, the sensor verified its flexibility, and the sensor can be used in wearable technologies.

**Figure 10:**
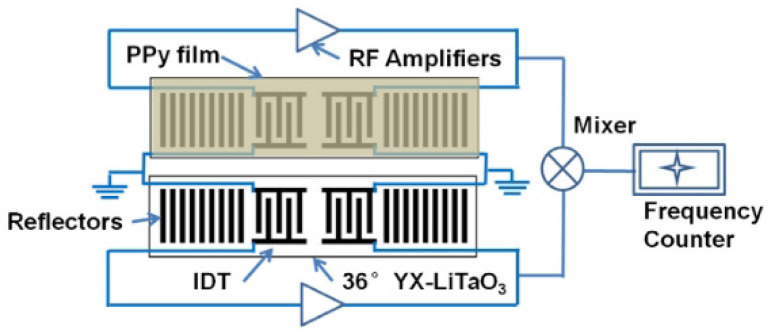
Schematic of the SAW-based sensor with PPy nanoparticles. (Reprinted with permission from [82]. Copyright 2013 Elsevier.

Mashat et al. presented a SAW-based gas sensor with PPy nanofibers [81]. The operating frequency of the SAW sensor was 90.6 MHz, and the gases were detected by the frequency shift. The detected gases were H${}_{2}$ and NO${}_{2}$. As a result, the sensor response was 20 kHz towards 1% of H${}_{2}$ and 4.5 kHz towards 2.1 ppm of NO${}_{2}$. Li et al. proposed the SAW-based acetone gas sensor with PPy nanoparticles, as shown in Figure 10 [82]. The SAW-based sensor was operated at 300 MHz, and the change in operating frequency was monitored for the detection of acetone. The nanoscale structure of PPy offered a high surface area and led to high sensitivity and fast response/recovery time. The sensor response was linear to the acetone concentration in the range from 5.5 ppm to 80 ppm. As a result, the frequency shift was 10.9 kHz with 80 ppm acetone. Penza et al. proposed the SAW-based ammonia sensor with a Langmuir-Blodgett PPy film at 42 MHz. Ammonia concentrations from 46 ppm to 10,000 ppm were detected by insertion loss [83] and phase [84]. When the sensor was exposed to 10,000 ppm ammonia, the variations in the insertion loss and phase were approximately 0.3 dB and 65 degrees, respectively. They also verified the sensor response when the ammonia concentration ranged from 18 ppm to 200 ppm by the variation in the phase [85]. It was demonstrated that the sensitivity was better at concentrations below 200 ppm but lower at high concentrations (≥200 ppm).

Yan et al. proposed the SAW-based sensor for the detection of NO${}_{2}$ at 123.56 MHz [86]. PPy was synthesized with titanium dioxide (TiO${}_{2}$) as the sensing material. NO${}_{2}$ concentrations of 15 ppm, 50 ppm and 100 ppm were detected by the frequency shift. The sensor response was 110 Hz when the sensor was exposed to 100 ppm NO${}_{2}$. Additionally, sensor responses based on both the PPy film and the PPy/TiO${}_{2}$ film to 100 ppm NO${}_{2}$ and H${}_{2}$S were measured. It was verified that the use of the PPy/TiO${}_{2}$ film can improve the selectivity of NO${}_{2}$ from H${}_{2}$S.

### 4.3. PEDOT-Based Radio-Frequency/Microwave Chemical Gas Sensors

Because PEDOT is insoluble in water, it cannot be applied in the inkjet printing method. However, it can be printing solution by polymerization in a water soluble electrolyte such as polystyrene sulfonate (PSS) [87]. PEDOT:PSS has advantages such as simple solution processing, high electrical sensitivity and stability. Thus, PEDOT:PSS was loaded onto various sensors and widely used for sensing materials.

Kudpun et al. proposed an interdigital resonator-based gas sensor with PEDOT:PSS [88]. The resonance frequency of the interdigital resonator was 3.584 GHz, and the sensor detected ammonia gas ranging from 50 ppm to 2000 ppm by the frequency shift. The sensor exhibits a resonant frequency of 3.584 GHz for 50 ppm and 3.622 GHz for 20,000 ppm. Jaruwongrungsee et al. also used printed PEDOT:PSS for relative humidity detection [89]. They utilized the quartz crystal microbalance (QCM), which is operated at 12 MHz. The relative humidity from 25% to 70% was detected by the frequency shift. The sensor responses depending on the number of printed PEDOT:PSS layers from 1 to 20 were verified. As a result, for 20 PEDOT:PSS printed layers, the variation in the frequency at 70% relative humidity was greater than 10 kHz. Manzari et al. utilized the PEDOT:PSS to the RFID antenna for the detection of relative humidity from 50% to 100% [90]. Three RFID antennas are designed for the loaded position of PEDOT:PSS, which have center frequencies of 850 MHz, 870 MHz and 880 MHz. The concentrations of relative humidity were detected by turn-on power and backscattered power. Abbasi et al. also loaded PEDOT:PSS onto a patch antenna at 2.65 GHz [91]. The relative humidity values of 40%, 70% and 100% were determined from the resonance frequency. As a result, the variation in the frequency shift was 115 MHz when the relative humidity changed from 40% to 100%.

**Figure 11:**
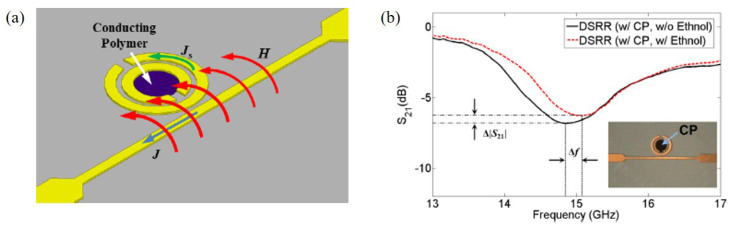
Ethanol gas sensor with DSRR and PEDOT:PSS. (**a**) Operation mechanism of the DSRR for gas detection using conducting polymer. (**b**) Transmission coefficients of the sensor with and without ethanol gas [92].

Kim et al. proposed an ethanol sensor based on a circular double split ring resonator (DSRR) loaded with PEDOT:PSS at 14.86 GHz [92]. When the end of the microstrip line was connected to the input source, a magnetic field was induced to the DSRR, as shown in Figure 11a. The PEDOT:PSS was coated inside the DSRR. The transmission coefficients of the sensor with/without ethanol gas are shown in Figure 11b. The 100 ppm ethanol gas was detected by the resonance frequency and transmission coefficient. According to the measured results, when the sensor was exposed to 100 ppm ethanol, the variations in the resonant frequency and transmission coefficient were 220 MHz and 0.79 dB, respectively.

**Figure 12:**
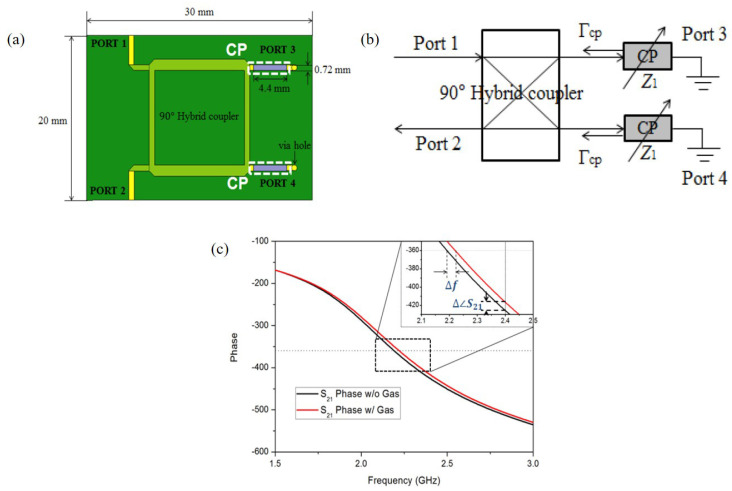
Ethanol gas sensor with hybrid coupler and PEDOT:PSS. (**a**) Schematic diagram of the sensor. (**b**) Equivalent circuit of the sensor. (**c**) Phase response of the sensor with and without ethanol gas [93].

They also applied the PEDOT:PSS to the hybrid coupler, which is operated at 2.4 GHz [93], as shown in Figure 12a. As shown in Figure 12b, the transmission line with the PEDOT:PSS can be represented as variable impedance Z${}_{1}$. When the ethanol gas molecules reacted with PEDOT:PSS, Z${}_{1}$ was changed due to adhesion of the ethanol gas molecules. Then, the reflection was changed, and 100 ppm ethanol gas could be detected by frequency and phase shift, as shown in Figure 12c. Consequently, when the sensor was exposed to 100 ppm ethanol gas, the variations in frequency and phase were 2.875 MHz and 9.09 degrees, respectively.

**Figure 13:**
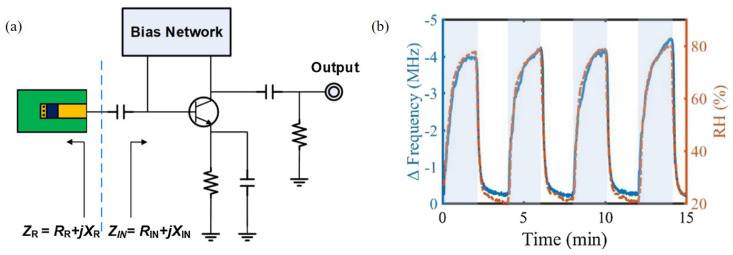
Characterization of the oscillator-based relative humidity sensor with PEDOT:PSS. (**a**) Schematic of the sensor. (**b**) Variation in oscillating frequency with change in relative humidity [94].

Kang et al. [94] designed an RF oscillator based on a negative resistance circuit with PEDOT:PSS at 916 MHz for relative humidity detection, as shown in Figure 13a. When PEDOT:PSS reacted with water vapor, both the conductance and capacitance of PEDOT:PSS increased. Thus, the oscillation frequency changed as the impedance of the sensing part varied. As shown in Figure 13b, the oscillation frequency decreased as the relative humidity increased. The variation in the oscillation frequency was 4.2 MHz when the relative humidity changed from 20% to 80%. Moreover, PEDOT:PSS reacted with water vapor in real time and showed outstanding results under the condition that the relative humidity varied dramatically with 1-min periods.

**Figure 14:**
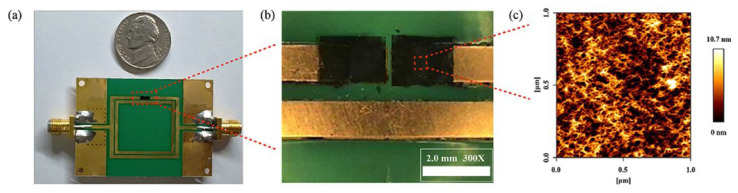
Characterization of the DSRR-based relative humidity sensor with PEDOT:PSS. (**a**) Photograph of the fabricated sensor. (**b**) Optical microscope image of the sensing region. (**c**) 2D AFM image of the PEDOT:PSS surface [95].

**Figure 15:**
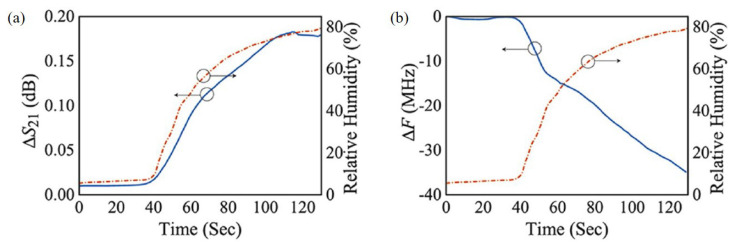
Measured results of the sensor. (**a**) Variation in transmission coefficient with change in relative humidity. (**b**) Variation in resonant frequency with change in relative humidity [95].

Park et al. proposed a relative humidity sensor loaded with PEDOT:PSS at 2.4 GHz [95]. The proposed sensor was based on the DSRR, as shown in Figure 14. PEDOT:PSS was deposited on both sides of the outer ring of the DSRR to enhance the sensitivity. Because PEDOT:PSS has high conductivity, it can replace the copper line of the DSRR without changing the characteristics of the resonator. As shown in Figure 14b,c, PEODT:PSS was well deposited on the PCB substrate with a roughness of less than 10.7 nm. The resonance frequency of the designed DSRR was 2.4 GHz, and the relative humidity ranging from 10% to 80% was detected by the resonance frequency shift and variation in the transmission coefficient. As shown in Figure 15, when the relative humidity increased from 10% to 80%, the variation in the transmission coefficient and resonance frequency shift were 0.254 dB and 42.1 MHz, respectively. The transmission coefficient increased because the chemical reaction between PEDOT:PSS and water vapor increased the conductivity of PEDOT:PSS. The resonance frequency decreased because the effective permittivity of PEDOT:PSS increased due to the high dielectric constant of the water vapor.

## 5. Summary and Perspective

In conclusion, CPs have been widely used as sensing materials for gas sensors in the microwave regime. The important parameters, such as backbone polymer, used microwave component, operating frequency, and detected gas, are summarized in Table 2.

However, further research and development of RF/microwave gas sensors using CPs should be conducted. First, the sensitivity of the sensors should be improved to ensure accuracy. By optimizing the component structure and operating frequency for gas sensing or integration with the active circuit, resolution of the sensor can be enhanced. Second, adaptation of the sensor to the RF system should be conducted. By integrating the sensor with the RF system, the sensor can transmit the signal by wireless. Moreover, it is cost-efficient and labor-saving. Downconverting the operating frequency to DC enables measurements without expensive instruments, such as vector network analyzers and spectrum analyzers. Finally, the CP-based sensor can be used as a flexible wearable sensor. Because of the flexibility of the sensor, the sensor can be attached to human skin or clothes without inconvenience.

## Figures and Tables

**Table 1:** Chemical structures of representative conducting polymers (CPs).

Polymer	Conductivity (S/cm)	Structure
Polyaniline (PANi)	30–200	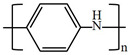
Polypyrrole (PPy)	10–7500	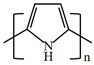
Poly(3,4-ethylenedioxythiophene) (PEDOT)	0.4–400	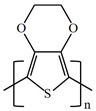
Polythiophene (PT)	10–1000	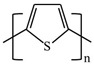
Polyacetylene (PA)	200–1000	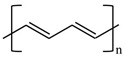
Poly(p-phenylene vinylene) (PPv)	1–1000	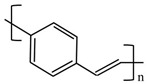
Poly(p-phenylene) (PPP)	500	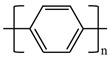
Poly(p-phenylene sulfide) (PPS)	3–300	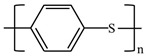
Polyisothianaphthene (PITN)	1–50	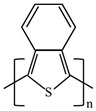

**Table 2:** Radio-Frequency/Microwave Chemical Gas Sensors Using Conducting Polymers.

Backbone Polymer	Component	Frequency	Detected Gas	Reference
PANi	SAW sensor	148 MHz	NH${}_{3}$	[74]
PANi	Interdigitated electrode	213.6 MHz	NH${}_{3}$	[72]
PANi	SAW sensor	146 MHz	Phosphorus-containing toxic gas	[76]
PANi	SAW sensor	107.2 MHz	H${}_{2}$	[78]
PANi	SAW sensor	107.2 MHz	H${}_{2}$, CO, NO${}_{2}$	[79]
PANi	Rayleigh SAW sensor	98.47 MHz	NO	[75]
PANi	SAW sensor	64.9 MHz	NO	[77]
PPy	RFID tag	940 MHz	NH${}_{3}$	[80]
PPy	SAW sensor	90.6 MHz	H${}_{2}$, NO${}_{2}$	[81]
PPy	SAW sensor	300 MHz	acetone	[82]
PPy	SAW sensor	42 MHz	NH${}_{3}$	[83,84,85]
PPy	SAW sensor	123.56 MHz	NO${}_{2}$	[86]
PEDOT	DSRR	14.86 GHz	ethanol	[92]
PEDOT	Interdigital resonator	3.584 GHz	NH${}_{3}$	[88]
PEDOT	Hybrid coupler	2.31 GHz	ethanol	[93]
PEDOT	DSRR	2.4 GHz	water vapor	[95]
PEDOT	Oscillator	916 MHz	water vapor	[94]
PEDOT	QCM	12 MHz	water vapor	[89]
PEDOT	RFID	850, 870, 880 MHz	water vapor	[90]
PEDOT	Antenna	2.65 GHz	water vapor	[91]
